# Supplementary material for: Safety and Tolerability of the BNT162b2 mRNA COVID-19 Vaccine in Dialyzed Patients. COViNEPH Project
Source: Medicina (Kaunas). 2021 Jul 19;57(7):732. doi: 10.3390/medicina57070732 (PMC8307559; doi:10.3390/medicina57070732)
Supplement: Supplementary file 1 [file medicina-57-00732-s001.zip › medicina-1254447-supplementary.pdf]

Form S1 Vaccine adverse effects questionnaire.

SAE 30 MINUTES AFTER VACCINATION

SOLICITED LOCAL REACTIONS

REDNESS[a]	NO	MILD	MODERATE	SEVERE	4 GRADE
SWELLING[a]	NO	MILD	MODERATE	SEVERE	4 GRADE
PAIN AT THE INJECTION SITE[b]	NO	MILD	MODERATE	SEVERE	4 GRADE

[a]Mild: >2,0 to 5,0cm; moderate: >5,0 to 10,0cm; severe: >10,0cm; grade 4: necrosis (redness or swelling categories) or exfoliative dermatitis (redness category only)

[b]Mild: does not interfere with activity; moderate: some interference with activity; severe: prevents daily activity; grade 4: emergency room visit or hospitalization for severe pain at the injection site

SOLICITED SYSTEMIC REACTIONS

FEVER[a]	NO	MILD	MODERATE	SEVERE	4 GRADE
FATIGUE[c]	NO	MILD	MODERATE	SEVERE	4 GRADE
HEADACHE[c]	NO	MILD	MODERATE	SEVERE	4 GRADE
CHILLS[c]	NO	MILD	MODERATE	SEVERE	4 GRADE
VOMITING[d]	NO	MILD	MODERATE	SEVERE	4 GRADE
DIARRHEA[e]	NO	MILD	MODERATE	SEVERE	4 GRADE
NEW OR WORSENING MUSCLE PAIN[c]	NO	MILD	MODERATE	SEVERE	4 GRADE
NEW OR WORSENING JOINT PAIN[c]	NO	MILD	MODERATE	SEVERE	4 GRADE
USE OF ANTYPYRETIC OR PAIN MEDICATION	YES / NO				

[a]Grade 1: ≥38,0°C to 38,4°C; grade 2: 38,4°C to 38,9°C; grade 3: 38,9°C to 40,0°C, grade 4: ≥40,0°C

[c]Mild: does not interfere with activity; moderate: some interference with activity; severe: prevents daily activity; grade 4: emergency room visit or hospitalization for severe fatigue, severe headache, severe chills, severe muscle pain, or severe joint pain

[d]Mild: 1 to 2 times in 24 hours; moderate: >2 times in 24 hours; severe: requires intravenous hydration; grade 4: emergency room visit or hospitalization for severe vomiting

[e]Mild: 2 to 3 loose stools in 24 hours; moderate: 4 to 5 loose stools in 24 hours; severe: 6 or more loose stools in 24 hours; grade 4: emergency room visit or hospitalization for severe diarrhea

SAE, AE 30 DAYS AFTER VACCINATION

Table S1. Solicited local reactions in the study and the control group.

LOCAL REACTIONS					
		STUDY GROUP (DIALYZED)	CONTROL GROUP	STUDY GROUP (DIALYZED)	CONTROL GROUP
		N= 189<br>n (%)	N= 160<br>n (%)	N= 187<br>n (%)	N= 160<br>n (%)
		**Adverse event after 1st dose**		**Adverse event after 2nd dose**	
Pain at the injection site[a]	Any	112 (59,3)	99 (61,9)	113 (60,4)	85 (53,1)
	Mild	99 (52,4)	77 (48,1)	102 (54,5)	63 (39,4)
	Moderate	10 (5,3)	18 (11,3)	8 (4,3)	16 (10,0)
	Severe	3 (1,6)	4 (2,5)	3 (1,6)	6 (3,8)
Swelling[b]	Any	7 (3,7) [c]	20 (12,5)	2 (1,1) [d]	22 (13,8)
	Mild	7 (3,7)	15 (9,4)	2 (1,1)	11 (6,9)
	Moderate	0 (0,0)	5 (3,1)	0 (0,0)	10 (6,3)
	Severe	0 (0,0)	0 (0,0)	0 (0,0)	1 (0,6)
Redness[b]	Any	5 (2,6) [d]	19 (11,9)	6 (3,2) [d]	21 (13,1)
	Mild	5 (2,6)	13 (8,1)	5 (2,7)	14 (8,8)
	Moderate	0 (0,0)	6 (3,8)	1 (0,5)	7 (4,4)

[a] Mild: does not interfere with activity; moderate: some interference with activity; severe: prevents daily activity; grade 4: emergency room visit or hospitalization for severe pain at the injection site

[b] Mild: >2,0 to 5,0cm; moderate: >5,0 to 10,0cm; severe: >10,0cm; grade 4: necrosis (redness or swelling categories) or exfoliative dermatitis (redness category only)

Significances: (gray fields indicate statistically significant differences): [c] - p=0.002; [d] - p< 0.001

Table S2. Solicited systemic reactions in the study and the control group.

SYSTEMIC REACTIONS					
		STUDY GROUP (DIALYZED)	CONTROL GROUP	STUDY GROUP (DIALYZED)	CONTROL GROUP
		N= 189 n (%)	N= 160 n (%)	N= 187 n (%)	N= 160 n (%)
		**Adverse event after 1st dose**		**Adverse event after 2nd dose**	
Fatigue[a]	Any	22 (11,6)	24 (15,0)	42 (22,5)	36 (22,5)
	Mild	20 (10,6)	19 (11,9)	32 (17,1)	28 (17,5)
	Moderate	2 (1,1)	4 (2,5)	6 (3,2)	8 (5,0)
	Severe	0 (0,0)	1 (0,6)	4 (2,1)	0 (0,0)
New or worsened joint pain[a]	Any	10 (5,3)	9 (5,6)	20 (10,7)	27 (16,9)
	Mild	7 (3,7)	6 (3,8)	10 (5,3)	17 (10,6)
	Moderate	2 (1,1)	2 (1,3)	8 (4,3)	8 (5,0)
	Severe	1 (0,5)	1 (0,6)	2 (1,1)	2 (1,3)
New or worsened muscle pain[a]	Any	9 (4,8)	12 (7,5)	18 (9,6) [e]	28 (17,5)
	Mild	5 (2,6)	9 (5,6)	9 (4,8)	16 (10,0)
	Moderate	3 (1,6)	2 (1,3)	8 (4,3)	8 (5,0)
	Severe	1 (0,5)	1 (0,6)	1 (0,5)	4 (2,5)
Headache[a]	Any	6 (3,2)	12 (7,5)	12 (6,4) [f]	22 (13,8)
	Mild	4 (2,1)	9 (5,6)	10 (5,3)	13 (8,1)
	Moderate	1 (0,5)	2 (1,3)	2 (1,1)	5 (3,1)
	Severe	1 (0,5)	1 (0,6)	0 (0,0)	4 (2,5)
Fever[c]	≥38,0°C	4 (2,1)	6 (3,8)	13 (7,0)	14 (8,8)
	Grade 1	4 (2,1)	5 (3,1)	10 (5,3)	9 (5,6)
	Grade 2	0 (0,0)	1 (0,6)	2 (1,1)	4 (2,5)
	Grade 3	0 (0,0)	0 (0,0)	0 (0,0)	0 (0,0)
	Grade 4	0 (0,0)	0 (0,0)	0 (0,0)	1 (0,6)
Chills[a]	Any	3 (1,6)	7 (4,4)	7 (3,7)	14 (8,8)
	Mild	3 (1,6)	6 (3,8)	5 (2,7)	7 (4,4)
	Moderate	0 (0,0)	1 (0,6)	2 (1,1)	4 (2,5)
	Severe	0 (0,0)	0 (0,0)	0 (0,0)	3 (1,9)
Diarrhea[d]	Any	1 (0,5)	0 (0,0)	3 (1,6)	2 (1,3)
	Mild	1 (0,5)	0 (0,0)	0 (0,0)	1 (0,6)
	Moderate	0 (0,0)	0 (0,0)	3 (1,6)	0 (0,0)
	Severe	0 (0,0)	0 (0,0)	0 (0,0)	1 (0,6)
Vomiting[e]	Any	0 (0,0)	0 (0,0)	3 (1,6)	1 (0,6)
	Mild	0 (0,0)	0 (0,0)	1 (0,5)	1 (0,6)
	Moderate	0 (0,0)	0 (0,0)	2 (1,1)	0 (0,0)

[a] Mild: does not interfere with activity; moderate: some interference with activity; severe: prevents daily activity; grade 4: emergency room visit or hospitalization for severe fatigue, severe headache, severe chills, severe muscle pain, or severe joint pain

[c] Grade 1: ≥38,0°C to 38,4°C; grade 2: 38,4°C to 38,9°C; grade 3: 38,9°C to 40,0°C, grade 4: ≥40,0°C

[d] Mild: 2 to 3 loose stools in 24 hours; moderate: 4 to 5 loose stools in 24 hours; severe: 6 or more loose stools in 24 hours; grade 4: emergency room visit or hospitalization for severe diarrhea

[e] Mild: 1 to 2 times in 24 hours; moderate: >2 times in 24 hours; severe: requires intravenous hydration; grade 4: emergency room visit or hospitalization for severe vomiting

Significances: (gray fields indicate statistically significant differences): [e] - p=0.038; [f] – p= 0.028.

Table S3. Solicited local reactions in subgroups according to age

LOCAL REACTIONS									
		STUDY GROUP (DIALYZED)		CONTROL GROUP		STUDY GROUP (DIALYZED)		CONTROL GROUP	
		18-55 N= 48 n (%)	>55 N= 141 n (%)	18-55 N= 50 n (%)	>55 N= 110 n (%)	18-55 N= 48 n (%)	>55 N= 139 n (%)	18-55 N= 50 n (%)	>55 N= 110 n (%)
		**Adverse event after 1st dose**				**Adverse event after 2nd dose**			
Pain at the injection site[a]	Any	42(87,5)[c]	70 (49,6)	37(74,0)[d]	62 (56,4)	38(79,2)[e]	75 (54,0)	31 (62,0)	54 (49,1)
	Mild	35 (72,9)	64 (45,4)	24 (48,0)	53 (48,2)	35 (72,9)	67 (48,2)	20 (40,0)	43 (39,1)
	Moderate	4 (8,3)	6 (4,3)	11 (22,0)	7 (6,4)	1 (2,1)	7 (5,0)	8 (16,0)	8 (7,3)
	Severe	3 (6,3)	0 (0,0)	2 (4,0)	2 (1,8)	2 (4,2)	1 (0,7)	3 (6,0)	3 (2,7)
Swelling[b]	Any	3 (6,3)	4 (2,8)	7 (14,0)	13 (11,8)	0 (0,0)	2 (1,4)	11 (22,0)	11 (10,0)
	Mild	3 (6,3)	4 (2,8)	5 (10,0)	10 (9,1)	0 (0,0)	2 (1,4)	5 (10,0)	6 (5,5)
	Moderate	0 (0,0)	0 (0,0)	2 (4,0)	3 (2,7)	0 (0,0)	0 (0,0)	5 (10,0)	5 (4,5)
	Severe	0 (0,0)	0 (0,0)	0 (0,0)	0 (0,0)	0 (0,0)	0 (0,0)	1 (2,0)	0 (0,0)
	4th grade	0 (0,0)	0 (0,0)	0 (0,0)	0 (0,0)	0 (0,0)	0 (0,0)	0 (0,0)	0 (0,0)
Redness[b]	Any	2 (4,2)	3 (2,1)	7 (14,0)	12 (10,9)	1 (2,1)	5 (3,6)	9 (18,0)	12 (10,9)
	Mild	2 (4,2)	3 (2,1)	4 (8,0)	9 (8,2)	1 (2,1)	4 (2,9)	5 (10,0)	9 (8,2)
	Moderate	0 (0,0)	0 (0,0)	3 (6,0)	3 (2,7)	0 (0,0)	1 (0,7)	4 (8,0)	3 (2,7)

[a] Mild: does not interfere with activity; moderate: some interference with activity; severe: prevents daily activity;grade 4: emergency room visit or hospitalization for severe pain at the injection site

[b] Mild: >2,0 to 5,0cm; moderate: >5,0 to 10,0cm; severe: >10,0cm; grade 4: necrosis (redness or swelling categories) or exfoliative dermatitis (redness category only)

Significances: (gray fields indicate statistically significant differences): [c] -p<0.001; [d]- p=0.033; [e]- p=0.002.

Table S4. Solicited systemic reactions in subgroups according to age

SYSTEMIC REACTIONS									
		STUDY GROUP (DIALYZED)		CONTROL GROUP		STUDY GROUP (DIALYZED)		CONTROL GROUP	
		18-55<br>N= 48<br>n (%)	>55<br>N= 141<br>n (%)	18-55<br>N= 50<br>n (%)	>55<br>N= 110<br>n (%)	18-55<br>N= 48<br>n (%)	>55<br>N= 139<br>n (%)	18-55<br>N= 50<br>n (%)	>55<br>N= 110<br>n (%)
		**Adverse event after 1st dose**				**Adverse event after 2nd dose**			
Fatigue[a]	Any	9 (18,8)	13 (9,2)	13(26,0) [e]	11 (10,0)	13 (27,1)	29 (20,9)	15 (30,0)	21 (19,1)
	Mild	8 (16,7)	12 (8,5)	11 (22,0)	8 (7,3)	10 (20,8)	22 (15,8)	10 (20,0)	18 (16,4)
	Moderate	1 (2,1)	1 (0,7)	2 (4,0)	2 (1,8)	3 (6,3)	3 (2,2)	5 (10,0)	3 (2,7)
	Severe	0 (0,0)	0 (0,0)	0 (0,0)	1 (0,9)	0 (0,0)	4 (2,9)	0 (0,0)	0 (0,0)
New or worsened joint pain[a]	Any	2 (4,2)	8 (5,7)	7 (14,0) [f]	2 (1,8)	10(20,8)[i]	10 (7,2)	15(30,0)[k]	12 (10,9)
	Mild	1 (2,1)	6 (4,3)	4 (8,0)	2 (1,8)	5 (10,4)	5 (3,6)	9 (18,0)	8 (7,3)
	Moderate	1 (2,1)	1 (0,7)	2 (4,0)	0 (0,0)	5 (10,4)	3 (2,2)	5 (10,0)	3 (2,7)
	Severe	0 (0,0)	1 (0,7)	1 (2,0)	0 (0,0)	0 (0,0)	2 (1,4)	1 (2,0)	1 (0,9)
New or worsened muscle pain[a]	Any	3 (6,3)	6 (4,3)	8 (16,0) [g]	4 (3,6)	10(20,8)[g]	8 (5,8)	15 (30,0)[l]	13 (11,8)
	Mild	1 (2,1)	4 (2,8)	6 (12,0)	3 (2,7)	5 (10,4)	4 (2,9)	8 (16,0)	8 (7,3)
	Moderate	2 (4,2)	1 (0,7)	1 (2,0)	1 (0,9)	5 (10,4)	3 (2,2)	5 (10,0)	3 (2,7)
	Severe	0 (0,0)	1 (0,7)	1 (2,0)	0 (0,0)	0 (0,0)	1 (0,7)	2 (4,0)	2 (1,8)
Headache[a]	Any	1 (2,1)	5 (3,5)	7 (14,0) [h]	5 (4,5)	5 (10,4)	7 (5,0)	12(24,0[m]	10 (9,1)
	Mild	1 (2,1)	3 (2,1)	5 (10,0)	4 (3,6)	5 (10,4)	5 (3,6)	8 (16,0)	5 (4,5)
	Moderate	0 (0,0)	1 (0,7)	1 (2,0)	1 (0,9)	0 (0,0)	2 (1,4)	3 (6,0)	2 (1,8)
	Severe	0 (0,0)	1 (0,7)	1 (2,0)	0 (0,0)	0 (0,0)	0 (0,0)	1 (2,0)	3 (2,7)
Fever[c]	≥38,0°C	1 (2,1)	3 (2,1)	3 (6,0)	3 (2,7)	7 (14,6) [j]	6 (4,3)	5 (10,0)	9 (8,2)
	Grade 1	1 (2,1)	3 (2,1)	3 (6,0)	2 (1,8)	5 (10,4)	5 (3,6)	3 (6,0)	6 (5,5)
	Grade 2	0 (0,0)	0 (0,0)	0 (0,0)	1 (0,9)	1 (2,1)	1 (0,7)	1 (2,0)	3 (2,7)
	Grade 3	0 (0,0)	0 (0,0)	0 (0,0)	0 (0,0)	1 (2,1)	0 (0,0)	0 (0,0)	0 (0,0)
	Grade 4	0 (0,0)	0 (0,0)	0 (0,0)	0 (0,0)	0 (0,0)	0 (0,0)	1 (2,0)	0 (0,0)
Chills[a]	Any	3 (6,3)	0 (0,0)	3 (6,0)	4 (3,6)	4 (8,3)	3 (2,2)	5 (10,0)	9 (8,2)
	Mild	3 (6,3)	0 (0,0)	3 (6,0)	3 (2,7)	3 (6,3)	2 (1,4)	3 (6,0)	4 (3,6)
	Moderate	0 (0,0)	0 (0,0)	0 (0,0)	1 (0,9)	1 (2,1)	1 (0,7)	1 (2,0)	3 (2,7)
	Severe	0 (0,0)	0 (0,0)	0 (0,0)	0 (0,0)	0 (0,0)	0 (0,0)	1 (2,0)	2 (1,8)
Diarrhea[d]	Any	0 (0,0)	1 (0,7)	0 (0,0)	0 (0,0)	1 (2,1)	2 (1,4)	1 (2,0)	1 (0,9)
	Mild	0 (0,0)	1 (0,7)	0 (0,0)	0 (0,0)	0 (0,0)	0 (0,0)	0 (0,0)	1 (0,9)
	Moderate	0 (0,0)	0 (0,0)	0 (0,0)	0 (0,0)	1 (2,1)	2 (1,4)	0 (0,0)	0 (0,0)
	Severe	0 (0,0)	0 (0,0)	0 (0,0)	0 (0,0)	0 (0,0)	0 (0,0)	1 (2,0)	0 (0,0)
Vomiting[e]	Any	0 (0,0)	0 (0,0)	0 (0,0)	0 (0,0)	1 (2,1)	2 (1,4)	0 (0,0)	1 (0,9)
	Mild	0 (0,0)	0 (0,0)	0 (0,0)	0 (0,0)	1 (2,1)	0 (0,0)	0 (0,0)	1 (0,9)
	Moderate	0 (0,0)	0 (0,0)	0 (0,0)	0 (0,0)	0 (0,0)	2 (1,4)	0 (0,0)	0 (0,0)

[a] Mild: does not interfere with activity; moderate: some interference with activity; severe: prevents daily activity; grade 4: emergency room visit or hospitalization for severe fatigue, severe headache, severe chills, severe muscle pain, or severe joint pain

[c] Grade 1: ≥38,0°C to 38,4°C; grade 2: 38,4°C to 38,9°C; grade 3: 38,9°C to 40,0°C, grade 4: ≥40,0°C

[d] Mild: 2 to 3 loose stools in 24 hours; moderate: 4 to 5 loose stools in 24 hours; severe: 6 or more loose stools in 24 hours; grade 4: emergency room visit or hospitalization for severe diarrhea

[e] Mild: 1 to 2 times in 24 hours; moderate: >2 times in 24 hours; severe: requires intravenous hydration; grade 4: emergency room visit or hospitalization for severe vomiting.

Significances: (gray fields indicate statistically significant differences): [e] -p=0.009; [f]- p=0.002; [g]- p=0.006; [h] - p=0.035; [i] - p=0.008; [j]- p=0.016; [k] - p=0.003; [l] - p=0.005; [m] - p=0.01.

Table S5. Solicited local reactions in subgroups according to gender.

LOCAL REACTIONS									
		STUDY GROUP (DIALYZED)		CONTROL GROUP		STUDY GROUP (DIALYZED)		CONTROL GROUP	
		Female N= 66 n (%)	Male N= 123 n (%)	Female N= 63 n (%)	Male N= 97 n (%)	Female N= 65 n (%)	Male N= 122 n (%)	Female N= 63 n (%)	Male N= 97 n (%)
		**Adverse event after 1st dose**				**Adverse event after 2nd dose**			
Pain at the injection site[a]	Any	43 (65,2)	69 (56,1)	47(74,6)[c]	52 (53,6)	44 (67,7)	69 (56,6)	43(68,3)[d]	42 (43,3)
	Mild	34 (51,5)	65 (52,8)	37 (58,7)	40 (41,2)	39 (60,0)	63 (51,6)	29 (46,0)	34 (35,1)
	Moderate	6 (9,1)	4 (3,3)	9 (14,3)	9 (9,3)	3 (4,6)	5 (4,1)	11 (17,5)	5 (5,2)
	Severe	3 (4,5)	0 (0,0)	1 (1,6)	3 (3,1)	2 (3,1)	1 (0,8)	3 (4,8)	3 (3,1)
Swelling[b]	Any	2 (3,0)	5 (4,1)	18 (28,6)	4 (4,1)	0 (0,0)	2 (1,6)	19 (30,2)	3 (3,1)
	Mild	2 (3,0)	5 (4,1)	14 (22,2)	3 (3,1)	0 (0,0)	2 (1,6)	10 (15,9)	1 (1,0)
	Moderate	0 (0,0)	0 (0,0)	4 (6,3)	1 (1,0)	0 (0,0)	0 (0,0)	9 (14,3)	1 (1,0)
	Severe	0 (0,0)	0 (0,0)	0 (0,0)	0 (0,0)	0 (0,0)	0 (0,0)	0 (0,0)	1 (1,0)
Redness[b]	Any	1 (1,5)	4 (3,3)	15 (23,8)	4 (4,1)	2 (3,1)	4 (3,3)	19 (30,2)	2 (2,1)
	Mild	1 (1,5)	4 (3,3)	10 (15,9)	3 (3,1)	1 (1,5)	4 (3,3)	12 (19,0)	2 (2,1)
	Moderate	0 (0,0)	0 (0,0)	5 (7,9)	1 (1,0)	1 (1,5)	0 (0,0)	7 (11,1)	0 (0,0)

[a] Mild: does not interfere with activity; moderate: some interference with activity; severe: prevents daily activity; grade 4: emergency room visit or hospitalization for severe pain at the injection site

[b] Mild: >2,0 to 5,0cm; moderate: >5,0 to 10,0cm; severe: >10,0cm; grade 4: necrosis (redness or swelling categories) or exfoliative dermatitis (redness category only)

Significances: (gray fields indicate statistically significant differences): [c] -p=0.008; [d]- p=0.002.

Table S6. Solicited systemic reactions in subgroups according to gender.

SYSTEMIC REACTIONS									
		STUDY GROUP (DIALYZED)		CONTROL GROUP		STUDY GROUP (DIALYZED)		CONTROL GROUP	
		Female N= 66 n (%)	Male N= 123 n (%)	Female N= 63 n (%)	Male N= 97 n (%)	Female N= 65 n (%)	Male N= 122 n (%)	Female N= 63 n (%)	Male N= 97 n (%)
		**Adverse event after 1st dose**				**Adverse event after 2nd dose**			
Fatigue[a]	Any	8 (12,1)	14 (11,4)	19 (30,2)	5 (5,2)	22(33,8)[f]	20 (16,4)	24(38,1)[f]	12 (12,4)
	Mild	7 (10,6)	13 (10,6)	14 (22,2)	5 (5,2)	19 (29,2)	13 (10,7)	20 (31,7)	8 (8,2)
	Moderate	1 (1,5)	1 (0,8)	4 (6,3)	0 (0,0)	2 (3,1)	4 (3,3)	4 (6,3)	4 (4,1)
	Severe	0 (0,0)	0 (0,0)	1 (1,6)	0 (0,0)	1 (1,5)	3 (2,5)	0 (0,0)	0 (0,0)
New or worsened joint pain[a]	Any	6 (9,1)	4 (3,3)	7 (11,1)	2 (2,1)	15(23,1)[g]	5 (4,1)	17(27,0)[j]	10 (10,3)
	Mild	4 (6,1)	3 (2,4)	5 (7,9)	1 (1,0)	6 (9,2)	4 (3,3)	10 (15,9)	7 (7,2)
	Moderate	1 (1,5)	1 (0,8)	2 (3,2)	0 (0,0)	7 (10,8)	1 (0,8)	6 (9,5)	2 (2,1)
	Severe	1 (1,5)	0 (0,0)	0 (0,0)	1 (1,0)	2 (3,1)	0 (0,0)	1 (1,6)	1 (1,0)
New or worsened muscle pain[a]	Any	6 (9,1)	3 (2,4)	9 (14,3)	3 (3,1)	13(20,0)[h]	5 (4,1)	18(28,6)[i]	10 (10,3)
	Mild	3 (4,5)	2 (1,6)	7 (11,1)	2 (2,1)	5 (7,7)	4 (3,3)	10 (15,9)	6 (6,2)
	Moderate	2 (3,0)	1 (0,8)	2 (3,2)	0 (0,0)	7 (10,8)	1 (0,8)	6 (9,5)	2 (2,1)
	Severe	1 (1,5)	0 (0,0)	0 (0,0)	1 (1,0)	1 (1,5)	0 (0,0)	2 (3,2)	2 (2,1)
Headache[a]	Any	4 (6,1)	2 (1,6)	8 (12,7)	4 (4,1)	7 (10,8)	5 (4,1)	16(25,4)[g]	6 (6,2)
	Mild	3 (4,5)	1 (0,8)	5 (7,9)	4 (4,1)	7 (10,8)	3 (2,5)	9 (14,3)	4 (4,1)
	Moderate	0 (0,0)	1 (0,80	2 (3,2)	0 (0,0)	0 (0,0)	2 (1,6)	4 (6,3)	1 (1,0)
	Severe	1 (1,5)	0 (0,0)	1 (1,6)	0 (0,0)	0 (0,0)	0 (0,0)	3 (4,8)	1 (1,0)
Fever[c]	≥38,0°C	2 (3,0)	2 (1,6)	2 (3,2)	4 (4,1)	6 (9,2)	7 (5,7)	7 (11,1)	7 (7,2)
	Grade 1	2 (3,0)	2 (1,6)	2 (3,2)	3 (3,1)	5 (7,7)	5 (4,1)	5 (7,9)	4 (4,1)
	Grade 2	0 (0,0)	0 (0,0)	0 (0,0)	1 (1,0)	1 (1,5)	1 (0,8)	2 (3,2)	2 (2,1)
	Grade 3	0 (0,0)	0 (0,0)	0 (0,0)	0 (0,0)	0 (0,0)	1 (0,8)	0 (0,0)	0 (0,0)
	Grade 4	0 (0,0)	0 (0,0)	0 (0,0)	0 (0,0)	0 (0,0)	0 (0,0)	0 (0,0)	1 (1,0)
Chills[a]	Any	3 (4,5)	0 (0,0)	5 (7,9)	2 (2,1)	5 (7,7)[l]	2 (1,6)	11(17,5)[k]	3 (3,1)
	Mild	3 (4,5)	0 (0,0)	4 (6,3)	2 (2,1)	4 (6,2)	1 (0,8)	5 (7,9)	2 (2,1)
	Moderate	0 (0,0)	0 (0,0)	1 (1,6)	0 (0,0)	1 (1,5)	1 (0,8)	4 (6,3)	0 (0,0)
	Severe	0 (0,0)	0 (0,0)	0 (0,0)	0 (0,0)	0 (0,0)	0 (0,0)	2 (3,2)	1 (1,0)
Diarrhea[d]	Any	1 (1,5)	0 (0,0)	0 (0,0)	0 (0,0)	0 (0,0)	3 (2,5)	1 (1,6)	1 (1,0)
	Mild	1 (1,5)	0 (0,0)	0 (0,0)	0 (0,0)	0 (0,0)	0 (0,0)	1 (1,6)	0 (0,0)
	Moderate	0 (0,0)	0 (0,0)	0 (0,0)	0 (0,0)	0 (0,0)	3 (2,5)	0 (0,0)	0 (0,0)
	Severe	0 (0,0)	0 (0,0)	0 (0,0)	0 (0,0)	0 (0,0)	0 (0,0)	0 (0,0)	1 (1,0)
Vomiting[e]	Any	0 (0,0)	0 (0,0)	0 (0,0)	0 (0,0)	1 (1,5)	2 (1,6)	0 (0,0)	1 (1,0)
	Mild	0 (0,0)	0 (0,0)	0 (0,0)	0 (0,0)	1 (1,5)	0 (0,0)	0 (0,0)	1 (1,0)
	Moderate	0 (0,0)	0 (0,0)	0 (0,0)	0 (0,0)	0 (0,0)	2 (1,6)	0 (0,0)	0 (0,0)

[a] Mild: does not interfere with activity; moderate: some interference with activity; severe: prevents daily activity; grade 4: emergency room visit or hospitalization for severe fatigue, severe headache, severe chills, severe muscle pain, or severe joint pain

[c] Grade 1: ≥38,0°C to 38,4°C; grade 2: 38,4°C to 38,9°C; grade 3: 38,9°C to 40,0°C, grade 4: ≥40,0°C

[d] Mild: 2 to 3 loose stools in 24 hours; moderate: 4 to 5 loose stools in 24 hours; severe: 6 or more loose stools in 24 hours; grade 4: emergency room visit or hospitalization for severe diarrhea

[e] Mild: 1 to 2 times in 24 hours; moderate: >2 times in 24 hours; severe: requires intravenous hydration; grade 4: emergency room visit or hospitalization for severe vomiting

Significances: (gray fields indicate statistically significant differences): [f]-p=0.009; [g]- p<0.001; [h]- p=0.004; [i]- p=0.003; [j]- p=0.006; [k]- p=0.002; [l]- p=0.037.
